# Genetic diversity, clinical uses, and phytochemical and pharmacological properties of safflower (*Carthamus tinctorius* L.): an important medicinal plant

**DOI:** 10.3389/fphar.2024.1374680

**Published:** 2024-05-10

**Authors:** Hao Cheng, Chenglong Yang, Pengliang Ge, Yi Liu, Muhammad Mubashar Zafar, Beibei Hu, Tong Zhang, Zengchun Luo, Siyu Lu, Qin Zhou, Abdul Jaleel, Maozhi Ren

**Affiliations:** ^1^ Institute of Urban Agriculture, Chinese Academy of Agricultural Sciences, Chengdu National Agricultural Science and Technology Center, Chengdu, China; ^2^ School of Agricultural Sciences, Zhengzhou University, Zhengzhou, China; ^3^ College of Plant Science and Technology, Huazhong Agricultural University, Wuhan, China; ^4^ Chengdu Florascape Technology Service Center, Chengdu, China; ^5^ Department of Integrative Agriculture, College of Agriculture and Veterinary Medicine, United Arab Emirates University, Al Ain, United Arab Emirates

**Keywords:** safflower, genetic diversity, medical applications, phytochemicals and pharmacological effects, space sickness

## Abstract

Safflower (*Carthamus tinctorius* L.), a member of the Asteraceae family, is widely used in traditional herbal medicine. This review summarized agronomic conditions, genetic diversity, clinical application, and phytochemicals and pharmacological properties of safflower. The genetic diversity of the plant is rich. Abundant in secondary metabolites like flavonoids, phenols, alkaloids, polysaccharides, fatty acids, polyacetylene, and other bioactive components, the medicinal plant is effective for treating cardiovascular diseases, neurodegenerative diseases, and respiratory diseases. Especially, Hydroxysafflor yellow A (HYSA) has a variety of pharmacological effects. In terms of treatment and prevention of some space sickness in space travel, safflower could be a potential therapeutic agent. Further studies are still required to support the development of safflower in medicine. Our review indicates that safflower is an important medicinal plant and research prospects regarding safflower are very broad and worthy of further investigation.

## 1 Introduction

Metabolic Syndrome (MetS), Coronary Heart Disease (CHD), hypertension, dysmenorrhoea, and amenorrhoea are typical public’s health problems. Specifically, MetS is a cluster of metabolic risk factors that lead to 84 Cardiovascular Diseases (CVD) and diabetes (Ruyvaran et al., 2022). As a common heart disease, CHD is the leading cause of morbidity and mortality world-wide and has been characterized as a chronic immunoinflammatory, fibroproliferative disease fueled by lipids (Dong et al., 2019; Shaya et al., 2022). Hypertension is a fatal yet preventable risk factor for cardiovascular disease and is responsible for the majority of cardiovascular mortality (Alpsoy, 2020). Dysmenorrhoea is the term for painful menstruation. It is a common gynecological complaint among female adolescents, which reduces the quality of women’s life and is still an important public health problem (Barcikowska et al., 2020). As for Amenorrhoea, it is regarded as a kind of menstrual disorder in a woman of reproductive age, due to an abnormality in the hypothalamic-pituitary-ovarian axis, anatomical abnormalities of the genital tract or functional causes (Ghadirkhomi et al., 2022). Nowadays, many medicinal plants and their derivatives have been traditionally used for the prevention and treatment of different types of diseases and disorders, with high healing but minor toxic side effects (Abuova et al., 2022). Among those plants, safflower is effective for some typical public health problems.

Safflower, which originates from Ethiopia, Afghanistan, and Arab countries, has a long history of cultivation and is widely distributed worldwide (Ruyvaran et al., 2022). This crop can provide commercial oil and natural pigments for the food area, which is of great industrial importance. Flavonoids, quinochalcones, alkaloids, polyacetylenes, fatty acids, proteins, lignans, steroids, and polysaccharides are compound families for safflower. Among these chemical components, flavonoids and quinochalcones are the main active components with various pharmacological effects (Ren et al., 2020; Adamska and Biernacka, 2021; Rodríguez-Félix et al., 2021). Besides, the flowers and seeds of safflower are rich in oils, which makes it a dryland oilseed crop yielding high quality edible oil (Mani et al., 2020). In some Asian countries, the plant’s petals and oil are used as medicine to treat diseases, including coronary heart disease, hypertension, dysmenorrhoea, and amenorrhoea (Zhang et al., 2016). Additionally, the leaf and stem of safflower (>80% of the total plant) are considered by-products of the oil industry in Mexico and other countries (Del-Toro-Sánchez et al., 2021).

In order to fully develop safflower in the future, we examined the current advancements in genetic diversity, clinical application, phytochemicals and pharmacological properties of safflower. The potential application in space disease was also emphasized in this paper.

## 2 Agronomic conditions of safflower cultivation

**FIGURE 1:**
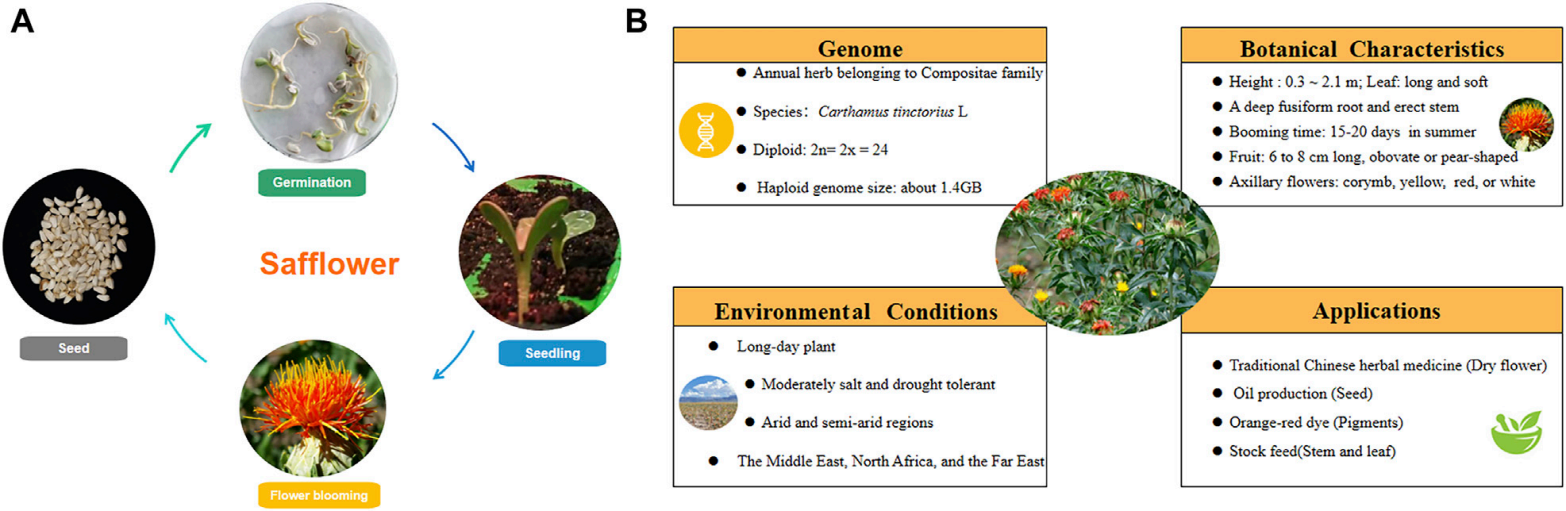
The biological characteristics and application of safflower. **(A)** the life cycle of safflower; **(B)** the characters of safflower.

The life cycle of safflower includes seed germination, seedling growth, flower blooming, and harvest. Information about the medicinal plant’s genome, botanical characteristics, growth conditions, and application is depicted in Figure 1. Safflower is considered a long-day plant, and most “spring” cultivars can be sown in late winter or early spring and flower rapidly in the absence of overwintering, dominating global safflower production. Some winter-hardy accessions that can be sown in autumn and survive winter conditions were also reported in Australia (Cullerne et al., 2021). As a promising oilseed crop with appreciable seed and oil yields, it has shown great suitability in arid regions mainly due to its high tolerance to cold, drought and soil salinity, which allows its cultivation in regions that experience dry spells like central Southern Italy, Brazilian Cerrado, and Xinjiang of China (de Oliveira Neto et al., 2022; Licata et al., 2023). The wide adaptation ability of safflower is associated with its deep root system, which may take up moisture and nutrients, especially nitrogen that has been leached below the rooting zone of most other crops, especially in sandy soils which are already deprived of essential plant nutrients (Hussain and Al-Dakheel, 2018). However, safflower is significantly sensitive to many soil and plant pathogens associated with wet conditions, with a particular aversion to wet soil during the period of germination (Joshi et al., 2021). In terms of irrigation, compared with normal irrigation, water shortage during the reproductive stage of safflower severely influenced production. Similarly, water stress could decline photosynthesis and crop nutrient uptake, decreasing safflower seed yield (Bijanzadeh et al., 2022). Temperature is a major environmental factor for safflower. During the rosette stage, seedlings of safflower exhibited good adaptability to temperature change and can tolerate even −5°C. In Sichuan, China, the optimum growth temperature is 15–18°C during the seedling stage (Li et al., 2022). Dormancy of safflower seeds is pronounced soon after removal from the parent plant. It was indicated that recently harvested seeds could efficiently germinate at 10°C in the dark, while seeds dry-stored at 20°C had low germination percentages (Silva et al., 2023). As for fertilizers, the application of plant growth promoting rhizobacteria (Azospirillum and *Azotobacter*) not only reduced the use of nitrogen and phosphate (NP) fertilizers up to 50%–75%, but also improved the seed protein and oil quality (Nosheen et al., 2016; Nosheen et al., 2018). Leaf miners, like *Liriomyza sativae* and *L. huidobrensis*, could cause yield loss and poor quality seriously for safflower. 2% emulsifiable concentrate, or the mixture of three insecticides (bifenthrin 20% water emulsions, thiamethoxam 25% water dispersible granule, abamectin 2% emulsifiable concentrate = 1:1:1) are suggested to spray on the plant’s leaves at squaring stage. Along with the safflower’s color turning from yellow to red, the flower could be harvested for different purposes. In the early stage of blooming, yellow pigment accumulates much, which can be used to prepare medicines and for soft drink dyeing; As the content of red pigments gradually increased in the middle stage, flowers are suggested to be harvested quickly and dried in the shade for the application of herbal medicine; While yellow parts are barely visible, safflower could be processed for dyeing fabrics chocolate, and cosmetics (Pu et al., 2019).

## 3 Genetic diversity of safflower germplasm resources

### 3.1 The origins and distribution of safflower

Safflower is best known as “kusum” (in India and Pakistan), a term derived from the Sanskrit word “kusumbha.” Meanwhile, this crop is known as “Golrang” in Iran and “honghua” (red flower) in China (Abuova et al., 2022). The use of safflower by humans dates back to ancient times; remains have been found at an archaeological site in Syria, believed to be from 7500 B.C. The wild ancestor of safflower is believed to be *Carthamus palestinus* Eig, and its domestication led to its cultivation in the Middle East, North Africa, India, and the Far East (Pearl et al., 2014; Ali et al., 2019; Ali et al., 2020). Phylogenetic analysis showed that safflower likely diverged from artichokes (*Cynara cardunculus*) and sunflowers (*Helianthus annuus*) approximately 30.7 and 60.5 million years ago, respectively (Wu et al., 2021).

Safflower was cultivated in Italy, France, and Spain as early as the Middle Ages (fifth to fifteenth centuries). The crop was first introduced to North America in the late 1890s and commercial cultivation began in the 1950s (Turgumbayeva et al., 2018). It was also introduced in northern Mexico and is becoming an increasingly attractive crop for farmers, which is cultivated for oil extraction purposes from seed (Del-Toro-Sánchez et al., 2021). According to the “Compendium of Materia Medica,” safflower was introduced to China via the Silk Road during the Han Dynasty (119 B.C.) and has been cultivated in China for more than 2,000 years. At present, the main production areas in China are Henan, Sichuan, Zhejiang, and Xinjiang Uyghur Autonomous Region. Among them, the safflower variety ‘Chuanhonghua’ is used as a medicinal herb in Sichuan, China (Liang and Wang, 2022). Good light conditions in Xinjiang make it the largest producer of safflower in China, accounting for more than 80% of the national production (Lin et al., 2022).

**FIGURE 2:**
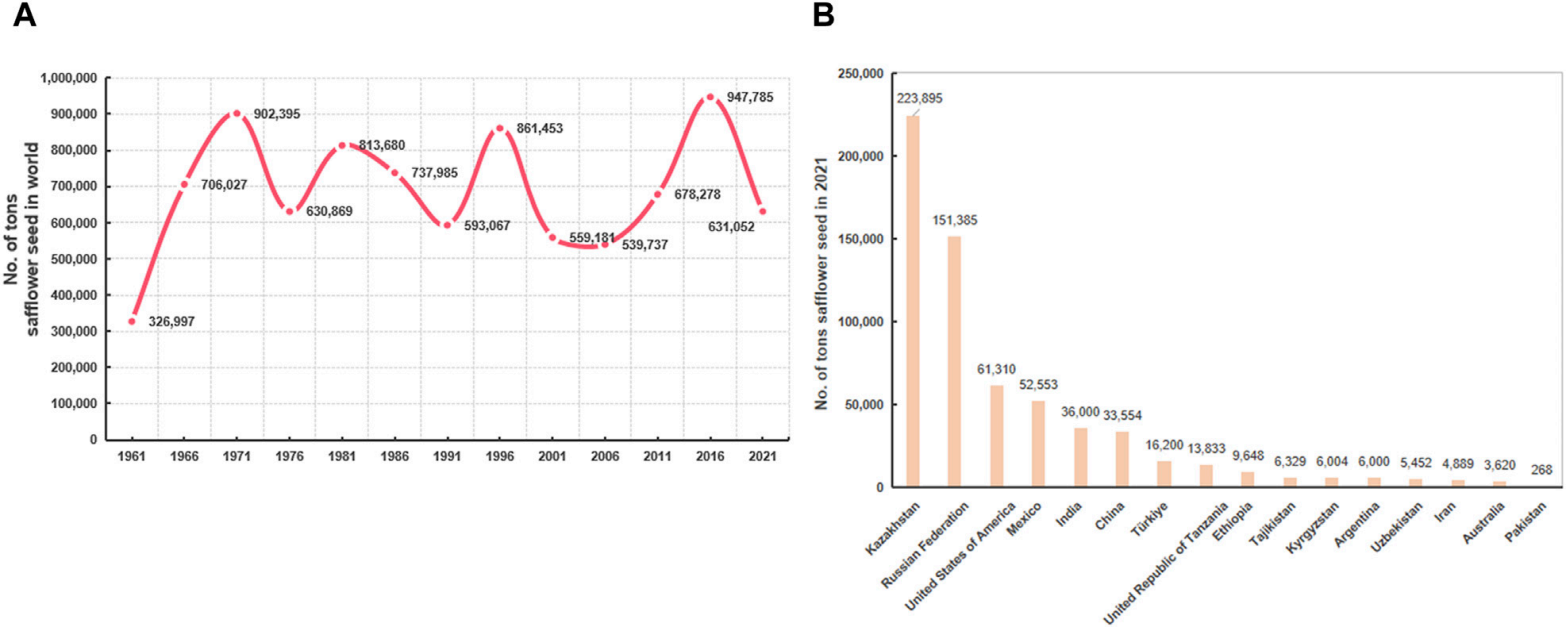
The distribution and yield of safflower around the world. **(A)** The changes in safflower yield in recent 60 years; **(B)** list of world’s top safflower-producing countries in 2021.

According to the Food and Agricultural Organization, the yield of safflower has increased over the past 60 years (Figure 2A). Almost 20 countries have cultivated safflower on a large scale, with a total planting area of 1,140,002 ha and an annual production of 948,516 tons in 2019 (Ali et al., 2019). In 2021, the global harvested safflower area was 850,431 ha, with safflower seed production of 631,051.6 tons. Kazakhstan accounted for the highest production of safflower seed (223,895.45 tons; area harvested, 43.27% of the global harvested area), followed by the Russian Federation, the United States of America, Mexico, India, China, and Turkey (Figure 2B).

### 3.2 Genetic diversity of safflower germplasm resources

#### 3.2.1 Genetic diversity for safflower with molecular markers

**FIGURE 3:**
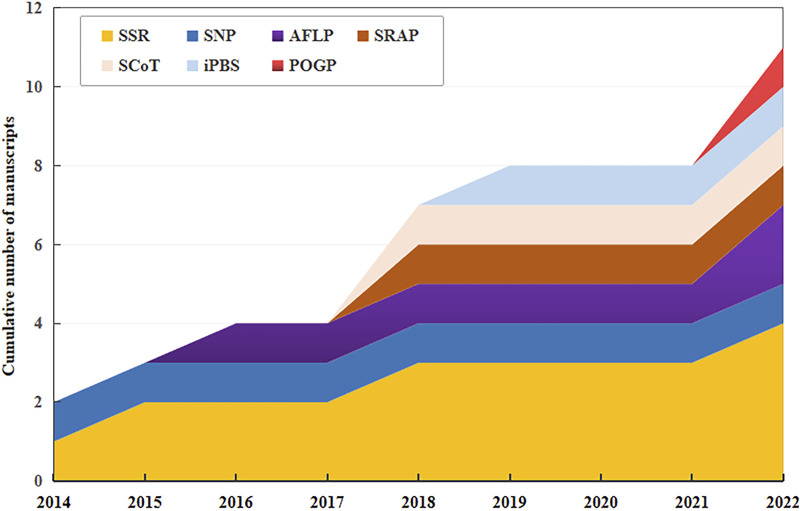
Cumulative number of manuscripts published between 2014 and 2022 reporting in the use of DNA molecular markers for safflower (*Carthamus tinctorius*) genotyping. SSR, simple sequence repeat; SNP, single-nucleotide polymorphism; AFLP, amplified fragment length polymorphism; SRAP, sequence-related amplified polymorphism; ScoT, start codon target; iPBS, inter-primary binding site; POGP, peroxidase gene polymorphism. The same as below.

Germplasm resources, which serve as repositories for diverse traits, are essential for crop improvement and industry development. Various DNA molecular markers, including simple sequence repeat (SSR), amplified fragment length polymorphism (AFLP), inter-simple sequence repeat (ISSR), expressed sequence tag SSR (EST-SSR), random amplified polymorphic DNA (RAPD), inter-primary binding site (iPBS)-retrotransposon, sequence-related amplified polymorphism (SRAP), and peroxidase gene polymorphism (POGP) have been used to assess the genetic diversity of safflower. The analysis has received considerable attention from the scientific community in the last decade (Figure 3).

Research led by scientists has revealed the rich genetic diversity of safflower (Table 1). 190 safflower individuals were genotyped using 133 single-nucleotide polymorphism (SNP) markers and identified previously undocumented genetic diversity. The results showed a modest reduction in gene diversity was observed in the commercial breeding lines (Pearl et al., 2014). Interestingly, most safflower breeding lines come from the Old World safflower germplasm (Lee et al., 2014).

**TABLE 1:** Genetic diversity characteristics of safflower with DNA molecular markers.

Sample locations	Sample numbers	Molecular markers	Primer numbers	He	Ho	I	PIC	*p* (%)	References
United States Department of Agriculture	190	SNP	133	0.26–0.27	0.04			81.2–82.71	Pearl et al. (2014)
Far East, India–Pakist and the Middle East	100	SSR	509	0.01–0.72	0.01–1.0		0.01–0.68		Lee et al. (2014)
Diverse agro-climatic zones of the world	23	SSR	93	0.04–0.62	0–0.95		0.3075	18.1–100	Ambreen et al. (2015)
Worldwide	531	AFLP	157			0.42–0.52			Kumar et al. (2016)
Different geographical regions of the world	100	SRAP, SCoT	23	0.21–0.29		0.22–0.42	0.1–0.68(SRAP) 0.12–0.49(SCoT)	18.1–100	Golkar and Mokhtari (2018)
Worldwide	124	SSR	93	0.016–0.76	0–0.958	0.02–0.73		64.5–97.8	Ambreen et al. (2018)
Iran and other countries	105	SSR	32				0.39		Golkar and Mokhtari (2018)
28 countries	131	iPBS	13	0.111–0.301		0.199–0.457	0.231–0.781		Ali et al. (2019)
28 countries	131	POGP	11			0.35–0.49	0.47–0.9		Yildiz et al. (2022)
Worldwide	84	AFLP, SSR	299			0.065–0.693			Singh et al. (2022)

Note: He, expected heterozygosity; Ho, observed heterozygosity; I, shannon information index; PIC, polymorphic information content. *p*, percent polymorphic loci.

Microsatellite markers (SSR) confirmed that 93 markers were polymorphic, and wild safflower species showed significant cross-species transfer (Ambreen et al., 2015). Using AFLP markers, the phenotypes of 531 representative safflower resources worldwide were evaluated for genetic diversity significant variation in agronomic traits was observed, with the highest variability and unique traits found in materials from the Indian subcontinent and from the America (Kumar et al., 2016). Genetic diversity analysis of 100 safflower genotypes from different geographical regions worldwide was performed using 12 polymorphic SRAPs and 11 polymorphic start codon target (SCoT) markers, showing that the genotypes of cultivated safflower were classified into the following five taxa: the Middle East (Iran, Iraq, Turkey, and Tajikistan), the Far East (India, Pakistan, and Korea), Europe, the American continent, and Africa (Egypt, Sudan, and Libya) (Golkar and Mokhtari, 2018). A safflower panel (CtAP) of 124 accessions from two core collections was analyzed using SSR markers. The Shannon diversity index (H = 0.7537) and Nei’s expected heterozygosity (I = 0.4432) revealed significant genetic diversity of CtAP (Ambreen et al., 2018). Additionally, 131 safflower germplasm resources from 28 countries were analyzed using 13 iPBS-retrotransposon markers, revealing the genetic diversity and population structure of seven inferred similarity centers (Ali et al., 2019). POGP markers were also used to analyse the genetic diversity of 131 safflower samples, which demonstrated that safflower germplasm resources collected from the Fertile Crescent region were clustered (Yildiz et al., 2022). Moreover, structural analysis of safflowers based on 155 AFLP and 144 SSR markers identified three main subpopulations (K = 3) with approximately 35% admixtures in the panel (Singh et al., 2022). In China, 13 local safflower varieties, including Chuanhonghua No. 2, Yunhonghua No. 2, and Xinhonghua No. 7, were breeded using safflower resources.

#### 3.2.2 Genetic diversity for safflower germplasm based on omics and biotechnology

There are some limits on the molecular marker information for evaluation of genetic diversity of safflower. Recently, With next-generation sequencing methods developing, omics and biotechnology have been used for investigating the genetic diversity of safflower (Table 2). 509 putative genomic SSR markers for sufficient genome coverage were acquired (Lee et al., 2014). Genetic mapping of millions of SNPs in safflower via whole-genome resequencing showed there were a total of 57,270 scaffolds, each containing five or more mapped SNPs (Bowers et al., 2016). A microarray based Diversity Array Technology (DArT) marker has been developed and used as an effective tool for genome diversity and population structure, and mapping construction in safflower. In-depth genome diversity analysis of worldwide diverse safflower accessions using NGS data generated by DArTseq technology was conducted, which confirmed the hypothesis that safflower was domesticated in the western Fertile Crescent and then spread to Africa and Europe (Hassani et al., 2020). Various mean genetic diversity parameters like expected heterozygosity (0.32) for 94 safflower accessions originating from 26 countries exhibited sufficient genetic diversity using 12232 silicoDArT markers. Interestingly, two DArTseq markers (DArT-45483051 and DArT-15672391) significantly associated with (*p* < 0.01) for 100-seed weight were identified, which may help develop high-yielding cultivars of safflower through marker-assisted breeding (Ali et al., 2020). cDNA-derived SSR markers are suitable for evaluation of genetic diversity. 35 SSR primer pairs were detected a high rate of polymorphism (>57%) among safflower accessions, physically mapped on safflower genome and could clearly discriminate the cultivated accessions from wild relatives (Ahmadi and Ahmadikhah, 2022). Additionally, a 1.17-Gb assembly with a contig N50 of 1.08 Mb was obtained for ‘Chuanhonghua 1’ using an integrated strategy combining Illumina, Oxford Nanopore, and Hi-C sequencing, 220 safflower lines were re-sequenced, resulting in the acquisition of a total of 7 402 693 high-quality SNPs (Chen et al., 2023). These studies showed that safflower germplasms had high genetic diversity, providing raw plant materials for alternative safflower breeding program.

**TABLE 2:** Genetic diversity of safflower with omics approaches.

Samples	Omics approaches	Genetic diversity	References
Accessions from Uzbekistan (No. 19), Korea (No. 44) and Mexico (No. 91)	454 pyrosequencing	509 putative genomic SSR markers for sufficient genome coverage were acquired	Lee et al. (2014)
96 F6 recombinant inbred lines of a cross between safflower and its wild progenitor	Whole-genome resequencing	A total of 57,270 scaffolds, each containing five or more mapped SNPs were obtained	Bowers et al. (2016)
89 safflower accessions from worldwide origins	DArTseq technology	3431 polymorphic DArTseq markers (1136 SilicoDArTs and 2295 SNPs) could be used for genetic diversity	Hassani et al. (2020)
94 safflower accessions collected from 26 countries	DArTseq technology	Various mean genetic diversity parameters exhibited sufficient genetic diversity using 12232 silicoDArT markers	Ali et al. (2020)
Safflower	Genic simple sequence repeats	1,841 SSR regions in 1,667 cDNA sequences were identified, and 35 SSR primer pairs could clearly discriminate the cultivated accessions from wild relatives	Ahmadi and Ahmadikhah (2022)
220 safflower lines	Whole-genome sequencing	A total of 7 402 693 high-quality SNPs were acquired	Chen et al. (2023)

## 4 The clinical application of safflower

Since long time, safflower has been used as a traditional herbal medicine in Asian countries (Table 3). In Iranian folklore medicine, safflower is an indispensable element for treating melancholy humor, vitiligo and black spots, rheumatism and paralysis, mouth ulcers, phlegm humor, numb limbs, diabetes, melancholia, dropsy, and the like. In Ayurveda, the herb is typically used for arthritis, scabies, and mastalgia. In Thailand, aqueous extract of safflower flowers is regarded as the hair color promoter, which has been largely used clinically (Delshad et al., 2018). Safflower is included as a traditional Chinese medicine in the Pharmacopoeia of the People’s Republic of China with the application of drugs for the treatment of amenorrhea, gastric tumors, as well as wounds. Especially, about 80 herbal medical products in Chinese Pharmacopoeia (2015 Ed.) are connected with safflower (Tong et al., 2021). Safflower has effects on relieving pain, dispersing blood stasis, and activating blood circulation, and a natural pigment named safflower yellow (SY) from safflower petals has been extensively applied in the medical field (Chen et al., 2022).

**TABLE 3:** Clinical application of safflower in some countries.

Countries	Types	Applications of safflower	References
Iran	Iranian folklore medicine	Treat diabetes, dropsy, melancholy humor, vitiligo and black spots, rheumatism and paralysis, psoriasis, poisoning, mouth ulcers, phlegm humor, and numb limbs	Delshad et al. (2018)
India	Ayurveda	Treat arthritis, scabies, and mastalgia	Delshad et al. (2018)
China	Traditional chinese medicine	Treat amenorrhea, gastric tumors, as well as wounds	Tong et al. (2021)
China	Modern medicine	Treat cardiovascular diseases	Turgumbayeva et al. (2018)
Thailand	Traditional Thai Medicine	Hair color promoter	Delshad et al. (2018)
Saudi Arabia	Modern medicine	Treat depression and anxiety	Albaiz (2022), Meneses et al. (2023)

Safflower preparations, including safflower injection, safflower yellow injection, and safflower soothing and revitalizing compresses, have been used clinically in modern medicine (Wu et al., 2021). For example, the water extract of safflower has been developed as an intravenous injection in China, which is extensively applied to treat cardiovascular diseases clinically (Turgumbayeva et al., 2018). In addition to treating physical diseases, a recent survey of 752 Saudis who had previously tried safflower for depression and anxiety showed that 279 (37.1%) reported that safflower was effective, whereas 389 (51.73%) reported some improvement (Albaiz, 2022). Consistent with the survey, a systematic review of scientific articles published between 2010 and 2020 showed that safflower flower extracts have an anxiolytic effect as effective as diazepam (Meneses et al., 2023). Due to its nutritional and healthy function, many safflower products, such as painkillers, health drinks, skin lotions, tablets, and other nutritional supplements, are currently on the market. The combination of safflower and other ingredients is effective in the treatment of some diseases. It was reported that GuHong injection, composed the safflower and chemical drug N-acetyl-L-glutamine, has great value in clinical for cerebrovascular diseases, such as ischemic stroke and related diseases (Wang et al., 2023a). Safflower and peach kernel herb-pair are widely used in traditional Chinese medicine for the treatment of liver fibrosis (Huang et al., 2023). The safflower-astragalus herbal pair has synergistic effects in the treatment of CHD (Yuan et al., 2023).

Some bioactive compounds of safflower is with great potential in clinical research. For example, Hydroxysafflor yellow A (HYSA), a natural compound from safflower, has a good effect of alleviating atherosclerosis, and clinical trials is needed (Xue et al., 2021). A meta-analysis with 31 groups containing a total of 2487 participants confirmed that HSYA can effectively treat diabetic kidney disease (DKD), which may provide therapies for DKD with new insights and promote its application in clinical practice (Fu et al., 2022). Besides, as unique organelles with a natural resilience to environmental stresses, safflower oleosomes may serve as a novel therapeutic agent in the field of dermatology, and human clinical research is required to determine their efficacy and safety (Patel et al., 2023).

## 5 Phytochemicals and pharmacological properties of safflower

### 5.1 Bioactive components of safflower

**FIGURE 4:**
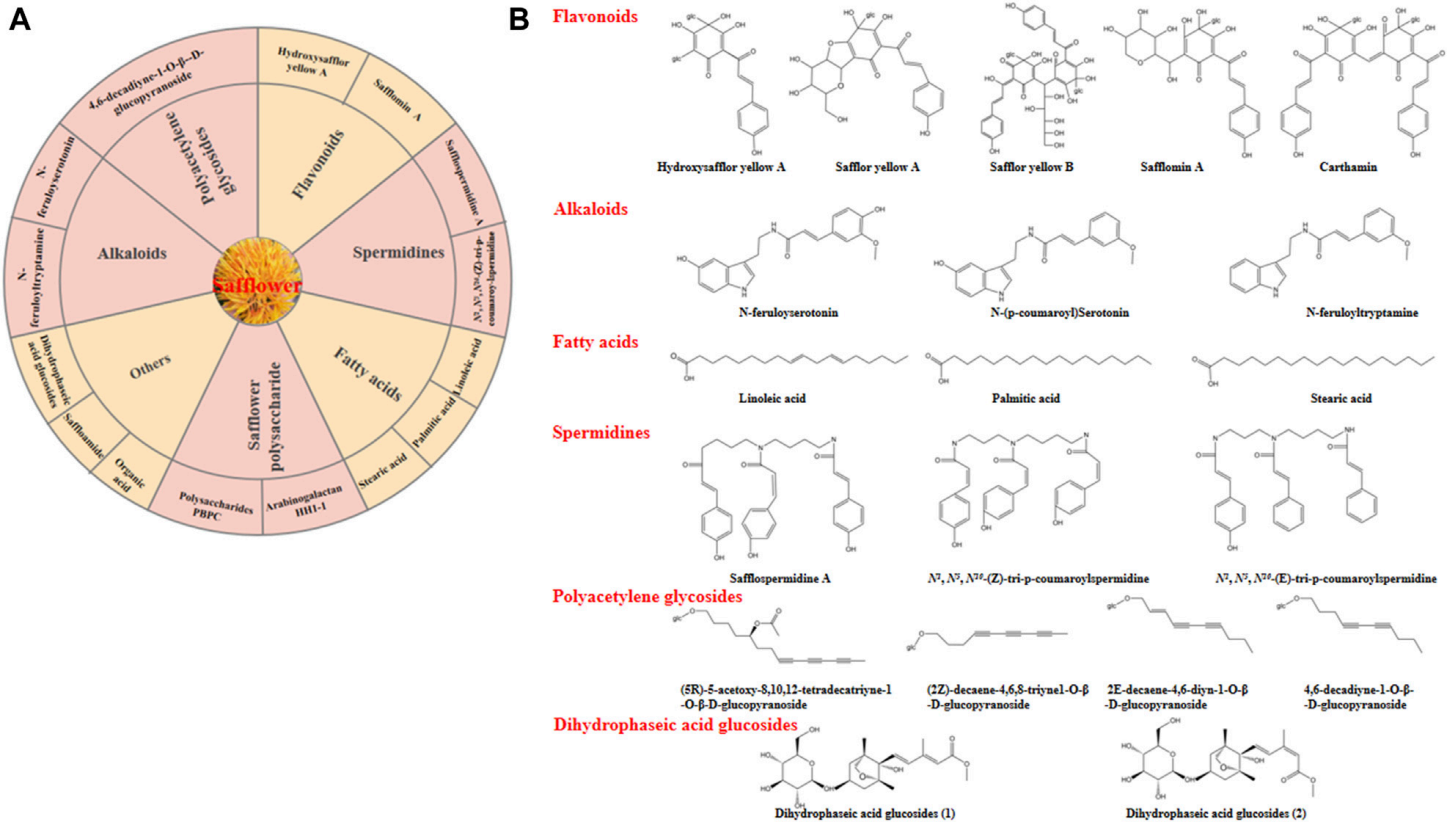
The main bioactive compounds of safflower. **(A)** some important bioactive compounds identified in safflower; **(B)** The chemical structures of the main bioactive compounds in safflower.

The obvious medical effects are associated with the chemical composition and pharmacological properties of safflower. In recent years, large amounts of bioactive components have been determined using various analytical methods, including liquid chromatography-mass spectrometry (LC-MS), gas chromatography-mass spectrometry (GC-MS), high-throughput metabolic fingerprinting, and nuclear magnetic resonance (NMR) (Mani et al., 2020). Safflower contains several bioactive components, such as vitamins A and E, polyunsaturated fatty acids (with linoleic acid being predominant at 70%), monounsaturated oleic acid (10%), small amounts of stearic acid, flavonoids, alkaloids, and polyalkenes (Liang and Wang, 2022). The main bioactive compounds and chemical structures of safflower were depicted in Figure 4.

#### 5.1.1 Flavonoids

Flavonoids and their glycosides (O- and C-glycosides) are an important group of phytochemicals in safflower. It is reported that the flavonoid content of safflower polyphenol extracts from flowers is 330 ± 23 catechin equivalent/100 g (Bacchetti et al., 2020). Previous studies have identified more than 104 compounds, among which six characteristic quinochalcone C-glycosides (QCGs), including HYSA and its two isomers, anhydrosafflor yellow B (AHSYB), safflomin C, and isosafflomin C, serve as biomarkers of safflower, suggesting that quinone chalcone and flavonoid compounds are active components (Si et al., 2016). Ultra-performance liquid chromatography coupled with triple-quadrupole linear ion-trap tandem mass spectrometry (UPLC-QTRAP^®^/MS2) analysis revealed that 16 components, including the signature components HYSA, AHSYB, safflomin C, and 13 other flavonoid glycosides, were related to the chromaticity characteristic of safflower (Pu et al., 2019). Similarly, ultra-high-performance liquid chromatography-quadrupole/time-of-flight mass spectrometry (UHPLC/Q-TOF-MS) analysis revealed that the flowers of safflower are rich in natural red and yellow pigments, which are flavonoid components such as safflomin A, safflomin C, and safflomin yellow B (Wang et al., 2021). A hybrid HDMSE-HDDDA method for non-targeted characterization identified 41 chalcones, 66 flavanols/flavones, 11 flavanones, six organic acids, one polyacetylene, and 16 others in safflower (Qian et al., 2022). UHPLC ESI-MS/MS analysis uncovered 212 flavonoid metabolites in safflower, including 64 flavones, 41 flavonols, 40 flavone-C-glycosides, 22 flavonones, 10 isoflavones, 10 catechin derivatives, 19 anthocyanins, 2 quinonechalcones, 2 flavonolignans, 1 alkaloid, and 1 proanthocyanidin (Ren et al., 2022). Recent studies have isolated a new flavonoid-saffloflavone, and six known compounds: kaempferol-3-O-rutinoside, kaempferol-3-O-sophoroside, quercetin-3-O-β-d-glucoside, quercetin-7-O-β-d-glucoside, luteolin-7-O-β-d-glucoside, and kaempferol-3-O-β-d-glucoside, from flowers of safflowers. All of these flavonoids can protect against H_2_O_2_-induced injury in H9c2 cells (Wang et al., 2022).

#### 5.1.2 Alkaloid and phenolic components

Alkaloids such as N-feruloyltryptamine and serotonin derivatives are widely distributed in the flowers of *C. tinctorius* and exhibit significant anticoagulant, hepatoprotective, neuroprotective, and antioxidant effects. Among them, 5-hydroxytryptamine derivatives, particularly N-(p-coumaroyl) serotonin and N-feruloylserotonin, are the main active alkaloids (Zhang et al., 2016). The main phenolic compounds of safflower seeds are N-feruloylserotonin-5-O-β-D-glucoside, 8-hydroxyarctigenin-4-O-β-D-glucoside, luteolin-7-O-β-D-glucoside, and N-feruloylserotonin (Mani et al., 2020).

#### 5.1.3 Safflower polysaccharides (SPSs)

Polysaccharides are the main bioactive components in safflower. A large number of soluble polysaccharides (SPS) from *C. tinctorius*, which have been proved to have antitumor, immunomodulatory, anti-cancer, and anti-diabetic effects (Zhou et al., 2018; Lin et al., 2022). HH1-1, an arabinogalactan with a relative molecular weight of 70.9 kDa, has been isolated from safflower flowers. The HH1-1 structure comprises a backbone of 1,6-linked Galp which is branched at C-3 position by a side chain of 1,3-linked Galp with sub-branches attached to the C-3 position (Yao et al., 2018). PBPC, a polysaccharide extracted from safflower bee pollen, has attracted the attention of the scientific community owing to its unique origin and biological activity (Wu et al., 2021). Four purified safflower polysaccharide fractions (named SSP1, SSP2, SSP3, and SSP4, respectively) were extracted from safflower by ultrasonic assisted extraction, among which SSP3 exhibited relatively higher antiproliferative activity, Fe^+3^-reduction activity, and ABTS+ scavenging activity (Wang et al., 2023b).

#### 5.1.4 Fatty acids

Oleic, linoleic, palmitic, and stearic acids are the most common fatty acids extracted from safflower. Safflower oil contains both saturated (palmitic, C16:0, and stearic C18:0) and unsaturated (oleic-C18:1, linoleic-C18:2, and linolenic-C18:3) fatty acids (Conte et al., 2016). The volatile oils from safflower variety ‘Ak-Mai’ of Kazakhstan were rich in undecanoic acid, heneicosanoic acid, octane, octadecanoic acid, 2-nonen-1-ol, 1.3-cyclohexadiene, myrtenoic acid, 1-eicosanol, hexcosane, and heptocosane (Turgumbayeva et al., 2018). The primary component of safflower seed oil is linoleic acid (84.48%), followed by palmitic acid (6.54%) and stearic acid (3.77%) (Alimi et al., 2022).

#### 5.1.5 Other active substances

In recent years, bioactive components, such as spermidine and polyacetylene, have been identified in safflower. Two new spermidine compounds (safflospermidine A and safflospermidine B) and two previously identified compounds (N1,N5,N10-(Z)-tri-p-coumaroylspermidine and N1,N5,N10-€-tri-p-coumaroylspermidine) were isolated from safflower (Zhang et al., 2016). In addition, Polyacetylene glucosides including (5R)-5-acetoxy-8,10,12-tetradecatriyne-1-O-β-D-glucopyranoside, (2Z)-decaene-4,6,8-triyne-1-O-β-D-glucopyranoside, (8Z)-1-[(3-O-β-D-glucosyl)-isovaleroyloxy]-8-decaene-4,6-diyne, (8Z)-decaene-1-isovaleroyloxy −4,6-diyne-10-O-β-D-glucopyranoside, and (2E,8E)-decadiene-4,6-diyne-1-O-β -D-glucopyranoside, were also identified from safflower buds (Li et al., 2021). A new flavonoid (saffloflavanside (**1**)), sesquiterpene (safflomegastigside (**2**)), and amide (saffloamide (**3**)), in addition to 22 known compounds from safflower were also isolated (Liu et al., 2022). Two dihydrophasic acid glycosides, dihydrophasic acid glucoside, which has anti-obesity effects, were also been isolated from *C. tinctorius* florets (Baek et al., 2020). Five new sesquiterpenoids (1–5) were isolated from the florets of safflower, which was named as (−)-(1R,4S,9S,11R)-caryophyll-8 (13)-en-14-ol-5-one (**1**), (+)-(1R,4R,9S,11R)-caryophyll-8 (13)-en-14-ol-5-one (**2**), (−)-(3Z,1R,5S,8S,9S,11R)-5,8-epoxycaryophyll-3-en-14-O-β-D-glucopyranoside (**3**), (+)-(1S,7R,10S)-guai-4-en-3-one-11-O-β-D-fucopyranoside (**4**), and (−)-(2R,5R,10R)-vetispir-6-en-8-one-11-O-β-D-fucopyranoside (**5**). These compounds have therapeutic effects for the treatment of atherosclerosis (Li et al., 2022).

### 5.2 Pharmacological activity of safflower

**FIGURE 5:**
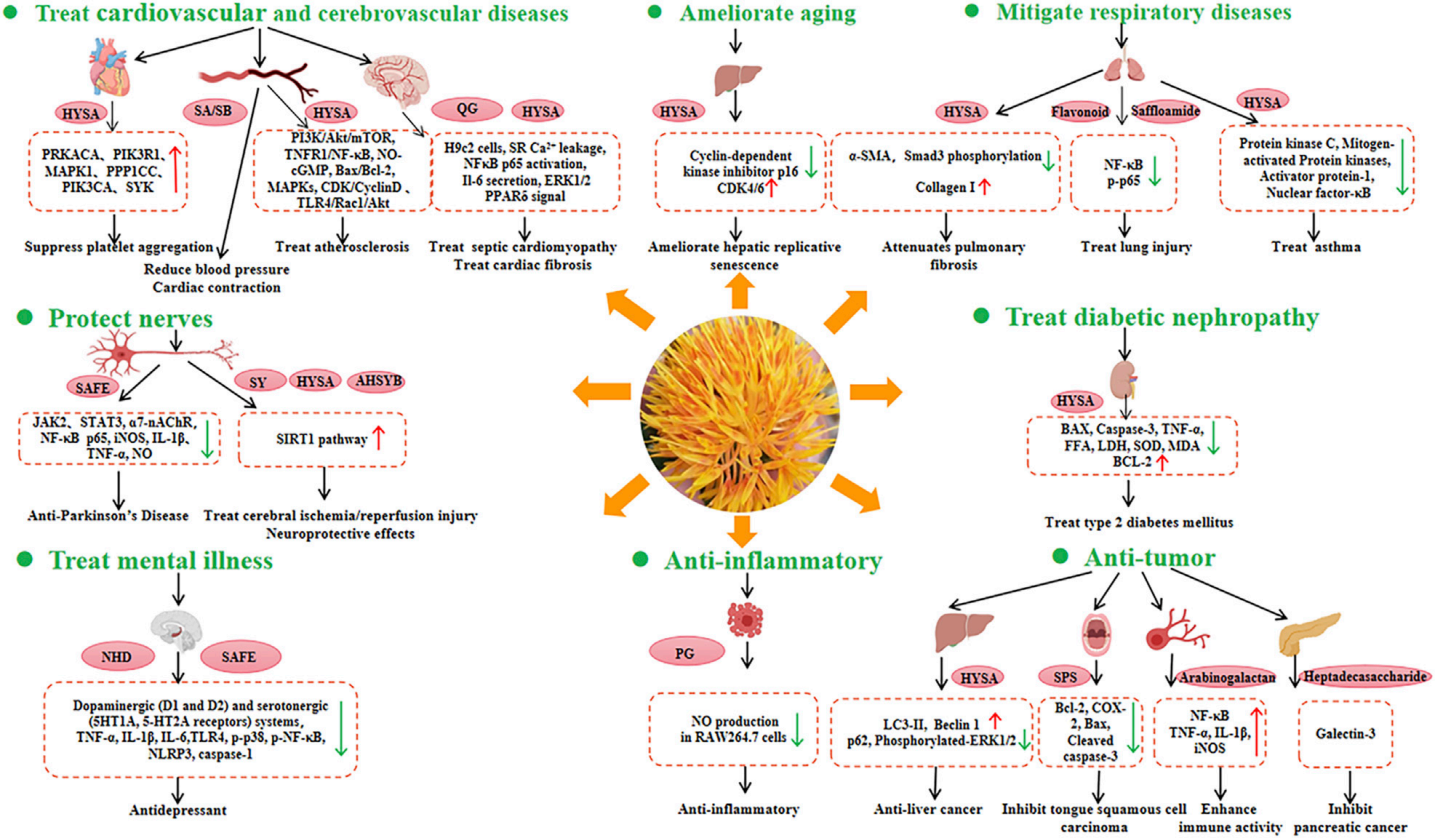
Health benefits and related molecular mechanisms of safflower. The red arrow represents increasing expression of genes, while the green arrow represents decreasing expression of genes. HYSA, Hydroxysafflor yellow A; SA, Safflomin A; SB, Safflomin B; QC, Quinochalcone C-Glycosides; SAFE, Safflower flavonoid extract; SY, Safflower yellow; AHSYB, Anhydrosafflor yellow B; NHD, N-Hexadecanoic acid; PG, Polyacetylene glucosides; SPS, Safflower polysaccharide.

Safflower is widely used in traditional Chinese medicine owing to its health benefits. It has a wide range of biological effects, including coronary artery dilation, alleviation of myocardial ischaemia, modulation of the immune system, and anticoagulant, antithrombotic, antioxidant, anti-aging, anti-hypoxic, anti-fatigue, anti-inflammatory, anti-liver fibrosis, anti-tumour, and analgesic activities (Figure 5).

#### 5.2.1 Treatment of cardiovascular and cerebrovascular diseases (CCVDs)

The incidence of CCVDs is increasing globally and constitutes a significant public health burden. Modern pharmacological studies have reported that safflower can be used to treat CCVDs. The therapeutic effects of hydroxysafflor yellow A (HYSA) on CCVD are due to its antioxidant, anti-inflammatory, and neuroprotective properties, which are mediated through complex signaling pathways (Wu et al., 2018; Bai et al., 2020). Safflower may regulate core genes (PRKACA, PIK3R1, MAPK1, PPP1CC, PIK3CA, and SYK) involved in the platelet activation pathway, potentially inhibiting platelet aggregation and having therapeutic effects in cardiovascular diseases (Yu et al., 2019).

In traditional Chinese medicine, safflower injection is used to improve blood circulation, because it can activate and promote blood circulation, as well as exert potent regulatory effects on the intrinsic coagulation system (Wang et al., 2018). HYSA has protective effects against atherosclerosis by regulating processes such as reverse cholesterol transport, fatty acid synthesis, oxidative stress, PI3K/Akt/mTOR, NLRP3 inflammasome, TNFR1/NF-κB, NO-cGMP, Bax/Bcl-2, mitogen-activated protein kinases (MAPKs), CDK/CyclinD, and TLR4/Rac1/Akt signaling pathways (Xue et al., 2021).

In terms of cardiomyopathy, hydroxysafflor yellow B and hydroxysafflor yellow C isolated from safflower florets have been shown to protect cultured H9c2 cardiomyocytes against H_2_O_2_-induced cytotoxicity by exerting antioxidant effects (Yue et al., 2014). Additionally, quercetin-7-O-β-d-glucoside, luteolin-7-O-β-d-glucoside, and kaempferol 3-O-β-d-glucoside have been found to protect H9c2 cells against H_2_O_2_-induced injury (Wang et al., 2022). *C. tinctorius* ethanolic extract was shown to alleviate lipopolysaccharide (LPS)-induced cardiac fibrosis through the ERK1/2 pathway, suggesting that safflower may be a potential cardioprotective agent against LPS-induced cardiac fibrosis (Han et al., 2017). One of the main functional components of safflower is HYSA. On the one hand, HYSA is a potential antioxidant with protective effects against myocardial injury, which can be attributed to its antioxidant effects (Yao et al., 2021). Moreover, it can enhance endurance performance by activating PPARδ signaling and promoting the utilisation of substrates in myocytes from glucose to fat (Sun et al., 2021).

#### 5.2.2 Neuroprotective effects

Parkinson’s disease (PD) and Alzheimer’s disease (AD) are neurodegenerative diseases. Safflower petal extracts have been shown free radical scavenging and neuroprotective effects (Abuova et al., 2022). The safflower flavonoid extract (SAFE) showed significant anti-PD effects, which might be due to the anti-inflammatory activity of flavonoids (Lei et al., 2020). Molecular docking analysis revealed that key components of SAFE, such as kaempferol-3-O-rutinoside or AHSYB, can bind to proteins such as TH, JAK2, STAT3, and α7-nAChR (Ablat et al., 2022). Thus, SAFE is a potential drug candidate for PD prevention.

SY and HYSA can protect nerves by alleviating amyloid β1-42-induced glutamate cycle disorder in an AD rat model and by improving synaptic structural plasticity, leading to enhanced learning and memory (Hou et al., 2020). In particular, HYSA can partially inhibit the expression of NF-κB p65 and iNOS, and downregulate the levels of IL-1β, TNF-α, and NO, leading to the suppression of inflammatory responses, attenuation of LPS-induced midbrain neurotoxicity and neuroinflammation, and alleviation of LPS-induced dopaminergic neuronal damage (Tiwari et al., 2018). Furthermore, HYSA and AHSYB may improve cell viability, decrease neuronal apoptosis, reduce infarct volume, improve neurological function, inhibite apoptosis, and reduce oxidative stress, which suggest that HYSA and AHSYB are potential drugs for the treatment of brain ischaemia/reperfusion (I/R) injury via the SIRT1 pathway (Fangma et al., 2021).

#### 5.2.3 Anti-respiratory disease activities

HYSA can exert various pharmacological effects, including preventive and therapeutic effects on some respiratory diseases such as acute lung injury and chronic obstructive pulmonary disease. Specifically, HYSA can mitigate the effects of bleomycin (BLM)-induced pulmonary fibrosis in mice (Jin et al., 2016). It also has the potential to be a novel drug for asthma owing to its ability to inhibit the upregulation of inflammatory factor expression, disruption of cellular barrier function, and suppression of the expression of protein kinase C, MAPK, activator protein-1, and nuclear factor-κB activation (Guo et al., 2018). Concerning lung injury, one new flavonoid (**1**), one new sesquiterpene (**2**), one new amide (**3**), and 22 known compounds (**4–25**) have been isolated from safflower flowers. Compounds **2–3**, **8–11**, and **15–19** demonstrated protective effects against LPS-induced BEAS-2B cell injury, suggesting that safflower could be developed as a drug for the treatment of lung injury (Liu et al., 2022).

#### 5.2.4 Anti-tumour and anti-inflammatory activities

Safflower contains various active ingredients that exhibit antitumor and anti-inflammatory effects (Zhou et al., 2018). HYSA induced autophagy in hepatocellular carcinoma cells by promoting Beclin 1 expression and inhibiting ERK phosphorylation, indicating HYSA may be a potential therapeutic agent for hepatocellular carcinoma (Chen et al., 2020). SPS is one of the most important active components of safflower and has been found to exert therapeutic effects on tongue squamous cell carcinoma by regulating the expression of Bcl-2, COX-2, Bax, and cleaved CASP3 (Zhou et al., 2018). Safflower arabinogalactan HH1-1 polysaccharide enhances immune activity by activating the NF-κB signaling pathway (Yao et al., 2018). Additionally, a highly branched heptadecasaccharide can target galectin-3 and inhibit pancreatic cancer cell growth (Hu et al., 2022). The polyacetylene glycosides (5R)-5-acetoxy-8,10,12-tetradecatriyne-1-O-β-D-glucopyranoside exhibited anti-inflammatory activity by inhibiting LPS-induced NO production in RAW264.7 cells (Li et al., 2021).

#### 5.2.5 Treatment of psychological disorders

Anxiety and depression are some of the causes of human suffering. However, the diagnosis and treatment remain challenging. Safflower can control monoamine transporters, resulting in the alleviation of neuropsychological damage. Moreover, safflower components (especially n-hexadecanoic acid) exhibit antidepressant-like effects by interacting with dopaminergic (D1 and D2) and serotonergic (5HT1A and 5-HT2A receptors) systems (Abbasi-Maleki and Mousavi, 2017). In addition, it can regulate the TLR4-NF-κB-NLRP3 signaling pathway, which contributes to its antidepressant effects (Chen et al., 2021). Clinically, Safflower has been applied to the Chinese herb DANSHEN-honghua (DSHH) to treat cognitive impairment after hippocampal ischaemic injury (Gu et al., 2018).

#### 5.2.6 Other bioactive activities

Safflower are found to have antiaging effects. HYSA is likely to regulate downstream genes such as CCNE1, CCNA2, P107, and MCM4, resulting in the amelioration of D-gal-induced hepatic replicative senescence (Min et al., 2020). The incidence of diabetic nephropathy (DN), a serious complication of diabetes, is the main cause of end-stage renal failure. HYSA was reported to show renal protective effects in high-fat diet-fed rats and streptozotocin-induced DN by inhibiting oxidative stress, attenuating the inflammatory response, and downregulating renal apoptosis. The results indicated the potential application of HYSA for the treatment of type 2 diabetes (Lee et al., 2020). Safflower by-products like leaf and stem are sources of obtaining phenolic compounds. Compounds from the by-products have been confirmed to have a protective effect against human erythrocytes, due to their ability to inhibit oxidative damage to erythrocyte membrane lipids as a result of free radicals (Del-Toro-Sánchez et al., 2021).

## 6 Potential applications of safflower in space medicine

The space environment alters human physiology, including fluid shifts, cardiovascular deconditioning, bone and muscle density loss, immune system dysregulation, and changes in the gastrointestinal tract as well as metabolic enzymes (Eyal and Derendorf, 2019). Cardiovascular system may be especially vulnerable due to the combined impacts of space radiation exposure, lack of gravity, and other spaceflight hazards (Huff et al., 2022). Long-term exposure to space microgravity also alters the time-frequency of heart rate in astronauts (Otsuka et al., 2016). Space motion sickness, which affects around 70% of astronauts, has symptoms of fatigue, vertigo, nausea, dizziness, headaches, vomiting, and cold sweating. It might cause potential problems for mission-critical tasks and astronauts’ wellbeing (Khalid et al., 2023). Additionally, astronauts also exhibit immune system dysregulation during space flight (Baba et al., 2020). Some possible hazards of space travel could be related to physical and psychological consequences on astronauts (Marazziti et al., 2022). Thus, medications are commonly administered during missions to treat space sickness.

**FIGURE 6:**
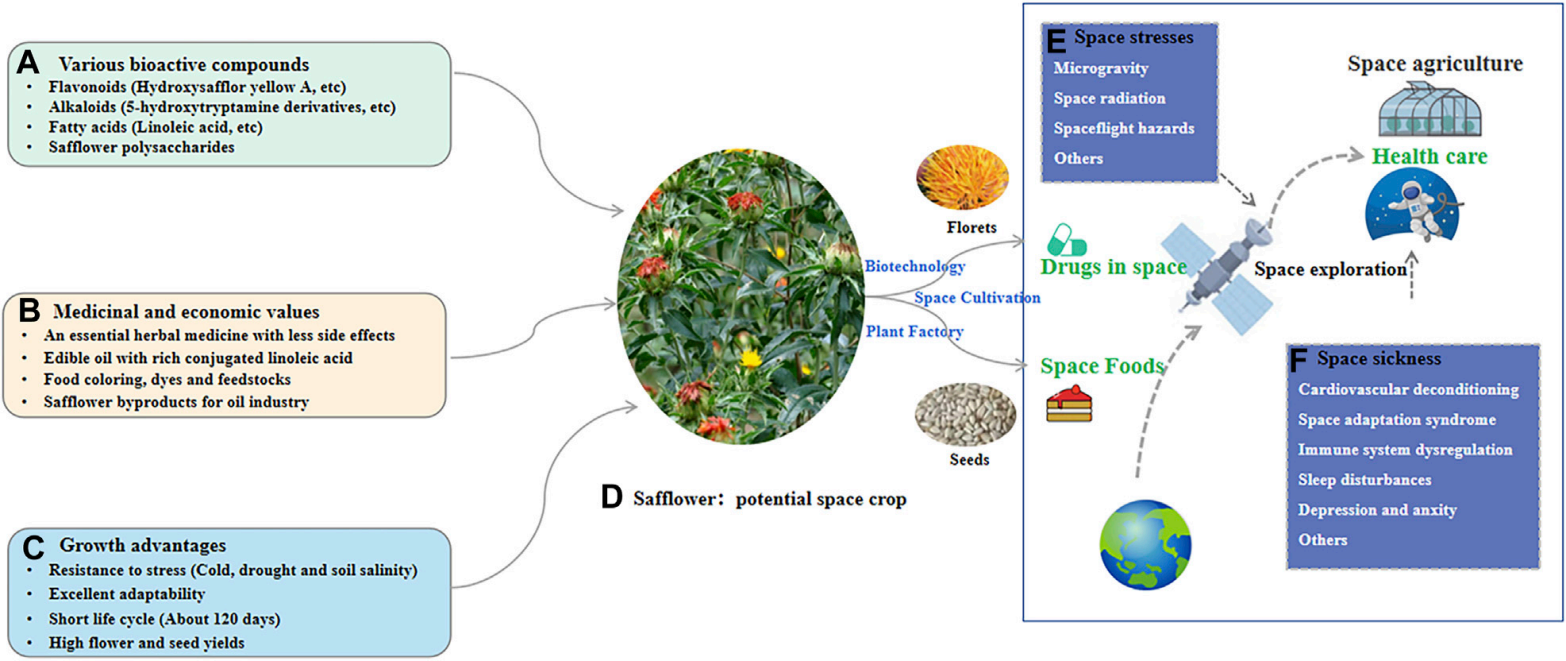
The potential application of safflower in space to supply space drugs and foods. **(A)** The bioactive compounds for safflower; **(B)** The medicinal and economic values for safflower; **(C)** The growth advantages for safflower; **(D)** The potential of safflower as space crop for providing both drugs and foods. **(E)** The main stresses for human in space; **(F)** Some space sickness during space exploration.

Safflower contains various active ingredients that exert pharmacological effects on the cardiovascular system, including ischemic myocardium-alleviating, anti-myocardial fibrosis, vascular protection, anti-thrombosis, lipid-lowering, antihypertensive, and anti-myocardial hypertrophic effects (Yu et al., 2019). Thus, the herb and its components could be used to treat some space sickness. Moreover, owing to anti-anxiety and antidepressant effects, the herb could be a potential therapeutic agent to alleviate psychological and psychopathological issues associated with space exploration. It was reported that an efficient space farming system is essential for human survival in space, and a whole-body edible and elite plant (WBEEP) strategy for space crop improvement is suggested (Liu et al., 2021). As safflower has advantages such as various bioactive compounds, medicinal values, and excellent adaptability, it could be WBEEP for providing medicine and food for human in space travel, with the application of biotechnology, plant factory, and cultivating strategy in space (Figure 6).

## 7 Conclusion and future perspectives

As a high-value medicinal plant with a wide range of genetic diversity, safflower is cultivated on a large scale in many countries, such as Kazakhstan, China, Russia, and the United States, where it is used to produce medicines, edible oils, food colouring agents, dyes, and fodders. Especially, it is rich in pharmacologically significant secondary metabolites, such as flavonoids, phenols, alkaloids, polysaccharides, and polyacetylene, with therapeutic effects on a variety of diseases, including cardiovascular diseases, neurodegenerative diseases, respiratory diseases, tumors, dysregulated inflammation, psychological diseases, aging, and diabetes. The phytochemistry and pharmacological activities of safflower have been investigated systematically in recent years. Considering its efficacy for the treatment of many physical and mental diseases, safflower could be herbal medicine for potential health challenges associated with space travel.

At present, the utilization of safflower relies on traditional planting and picking, which is low yield, labor cost, and easily affected by weather. As synthetic biology is a kind of biotechnology for efficient production of active ingredients, it is suggested to express key enzymes related to medicinal components in other organism like potato, yeast, and microalgae. Moreover, nanotechnology has shown promising advancements in the field of drug development and its delivery, with an opportunity to achieve better-targeted delivery, effective treatment, and an improved safety profile. It is suggested to apply nanotechnology for the controlled release of safflower components clinically. Although safflower has been recognized for its medicinal value, there have been limited studies on breeding, pharmacology, synthetic biology, and drug development. Hence, further research may focus on the following aspects: (1) utilizing the safflower germplasm resources to breed new safflower varieties with biotechnology; (2) understanding the phytochemistry and pharmacology of safflower, and exploring its new therapeutic potential; (3) uncovering the molecular mechanisms of safflower’s bioactive components synthesis, and conducting biosynthesis of pharmacologically active secondary metabolites of safflower by microorganisms like yeast and microalgae; (4) developing nanotechnology-based therapeutic agents with safflower and evaluating their efficacy and safety; (5) advancing medical research and clinical trial of safflower in treating and preventing space sickness.

## Figures and Tables

**Table:** 

**ACC**	Aminocyclopropane carboxylic acid
**ACO**	ACC oxidase
**AD**	Alzheimer’s disease
**AFLP**	Amplified fragment length polymorphism
**AHSYB**	Anhydrosafflor yellow B
**CCVDs**	Cardiovascular and cerebrovascular diseases
**CHD**	Coronary heart disease
**CVD**	Cardiovascular diseases
**DArT**	Diversity array technology
**DKD**	Diabetic kidney disease
**DN**	Diabetic nephropathy
**DSHH**	DANSHEN-honghua
**EST-SSR**	Expressed sequence tag SSR
**FCEtOH**	Carthami flos ethanolic extract
**GC-MS**	Gas chromatography-mass spectrometry
**HCT**	Hydroxycinnamoyl transferase
**HO**	High oleic
**HYSA**	Hydroxysafflor yellow A
**Ipbs**	Inter-primary binding site
**ISSR**	Inter-simple sequence repeat
**LC-MS**	Liquid chromatography-mass spectrometry
**LPS**	Lipopolysaccharide
**MAPKs**	Mitogen-activated protein kinases
**MetS**	Metabolic Syndrome
**NMR**	Nuclear magnetic resonance
**NP**	Nitrogen and phosphate
**PD**	Parkinson’s disease
**POGP**	Peroxidase gene polymorphism
**QCGs**	Quinochalcone C-glycosides
**RAPD**	Random amplified polymorphic DNA
**SAFE**	Safflower flavonoid extract
**SCoT**	Start codon target
**SNP**	Single-nucleotide polymorphism
**SPS**	Soluble polysaccharides
**SPSs**	Safflower polysaccharides
**SRAP**	Sequence-related amplified polymorphism
**SSR**	Microsatellite markers
**SSR**	Simple sequence repeat
**SY**	Safflower yellow
**UHPLC/Q-TOF-MS**	Ultra-high-performance liquid chromatography-quadrupole/time-of-flight mass spectrometry
**WBEEP**	Whole-body edible and elite plant
**WGD**	Whole genome duplication
